# Potential biological roles of exosomal non‐coding RNAs in breast cancer

**DOI:** 10.1096/fj.202500022R

**Published:** 2025-03-13

**Authors:** Xiang Li, Junyi Gong, Xiang Ni, Junli Yin, Yi Zhang, Zheng Lv

**Affiliations:** ^1^ Cancer Center The First Affiliated Hospital of Jilin University Changchun Jilin China

**Keywords:** breast cancer, exosomal ncRNAs, exosomes, non‐coding RNAs

## Abstract

Breast cancer (BC) is one of the most common malignant tumors among women, accounting for 24.5% of all cancer cases and leading to 15.5% of cancer‐related mortality. The treatment of BC patients remains a significant challenge due to the disease's high invasiveness, elevated metastatic potential, substantial drug resistance, and high recurrence rate. Exosomes, which are lipid‐bilayer extracellular vesicles ranging in size from 30 to 150 nm, mediate intercellular communication between tumor cells and surrounding cells in the tumor microenvironment by transferring various bioactive substances, such as proteins, lipids, and nucleic acids. Recently, growing evidence has demonstrated that non‐coding RNAs (ncRNAs) are enriched in exosomes and play a critical role in regulating cell proliferation, metastasis, drug resistance, and angiogenesis in BC. Consequently, exosomal ncRNAs have emerged as a promising therapeutic target for BC treatment, given their involvement in multiple processes of cancer progression. This review provides a comprehensive and in‐depth analysis of emerging exosomal ncRNAs in BC, highlighting their potential biological mechanisms and advanced applications in BC treatment.

## Graphical abstract

ncRNAs are enriched in exosomes and play a critical role in regulating cell proliferation, metastasis, drug resistance, and angiogenesis in BC. Consequently, exosomal ncRNAs have emerged as a promising therapeutic targets for BC treatment, given their involvement in multiple processes of cancer progression.

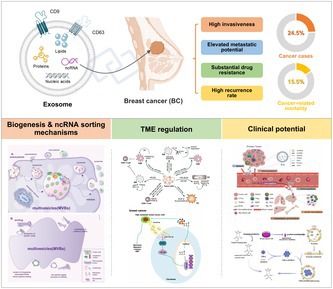

## 1 INTRODUCTION

According to the International Agency for Research on Cancer (IARC), breast cancer (BC) became the most common cancer globally in 2020, following lung cancer, with 2.26 million new cases diagnosed worldwide. It is estimated that by 2030, the incidence of BC in China will rise to 234,000 cases, marking a 31.15% increase compared to 2008.^1^ BC is categorized into four distinct subtypes—luminal A, luminal B, HER2‐positive, and basal‐like—based on the expression of specific receptors, including estrogen receptor (ER), progesterone receptor (PR), and human epidermal growth factor receptor 2 (HER2).^2^, ^3^, ^4^ Despite significant advancements in early detection, diagnosis, and treatment strategies (e.g., mastectomy, radiotherapy, and chemotherapy), BC remains a leading cause of cancer‐related deaths in women, largely due to poor prognosis associated with tumor metastasis, recurrence, and drug resistance.^5^, ^6^ Over the past few decades, numerous studies have sought to elucidate the underlying mechanisms driving BC occurrence, proliferation, and metastasis; however, many aspects remain unclear.^7^ Therefore, identifying key molecules and mechanisms is crucial for developing predictive and diagnostic biomarkers, as well as innovative therapeutic strategies to combat BC.

Exosomes, naturally occurring bilayer lipid vesicles ranging from 30 to 150 nm in diameter, are secreted by a wide variety of cell types, including both cancerous and normal cells, into surrounding environments such as blood, urine, saliva, and breast milk.^8^, ^9^ These exosomal components act as mediators of local and systemic cell‐to‐cell communication, regulating the biological functions of recipient cells by transferring intracellular contents, including messenger RNAs (mRNAs), lipids, DNA, ncRNAs, and other chemical messengers.^10^, ^11^ Common exosomal biomarkers are associated with the biogenesis, release, and fusion events of extracellular vesicles, including conserved proteins (transferrin receptor, clathrin, and caveolin) and tetraspanins (CD9, CD63, CD82, and CD81).^12^ Recently, increasing evidence has shown that tumor‐derived exosomes are crucial in the progression of malignant tumors, participating in processes such as tumor growth, metastasis, angiogenesis, and drug resistance.^13^ For instance, breast cancer (BC)‐derived exosomes can enhance the proliferation, motility, and metastatic capabilities of BC cells, thereby intensifying the oncogenic phenotype.^14^ In addition, Piao et al. demonstrated that BC cell‐derived exosomes stimulate macrophage polarization, thereby creating favorable conditions for lymph node metastasis in triple‐negative breast cancer (TNBC).^15^

The outstanding functionality and diversity of ncRNAs have attracted substantial interest in BC research. ncRNAs, despite their limited protein‐coding potential, are transcribed into various RNA species that play crucial roles in posttranscriptional regulation.^16^ Dysregulation of ncRNAs has been shown to drive the progression of BC by promoting proliferation, invasion, metastasis, and tumor cell cachexia.^17^ The growing interest in non‐coding RNAs (ncRNAs) has led to the development of microRNAs, circular RNAs, and long non‐coding RNAs.^18^, ^19^ miRNAs, a class of small ncRNAs ranging from 19 to 24 nucleotides in length, regulate gene expression posttranscriptionally by binding to complementary sites in the 3′‐untranslated region (UTR) of target mRNAs, leading to either translational repression or mRNA degradation.^20^ lncRNAs are broadly defined as ncRNA transcripts exceeding 200 nucleotides in length that are involved in gene regulation.^21^ circRNAs are ncRNAs generated by back splicing, characterized by a covalent single‐stranded loop structure, abundant miRNA‐binding sites, and the ability to act as miRNA sponges within cells.^22^ Importantly, ncRNAs can be packaged into extracellular vesicles, particularly exosomes, facilitating local or systemic transmission between cells. This capability enables the transfer of ncRNAs from tumor cells to normal cells, with various ncRNAs being synergistically packaged into tumor‐derived exosomes.^23^

Considering the critical role of exosomal ncRNAs in modulating cancer cell phenotypes both locally and systemically through the exosome‐dependent exchange of genetic information, we have synthesized recent findings on the functions and mechanisms of tumor‐derived exosomal miRNAs, lncRNAs, and circRNAs in breast cancer (BC). This review focuses on their involvement in BC cell proliferation, metastasis, and immunoregulation, with particular attention to the clinical implications of these exosomal ncRNAs in BC diagnosis and drug resistance. A comprehensive understanding of exosomal ncRNAs in BC is essential for gaining deeper insights into BC oncogenesis and progression, ultimately facilitating the development of novel therapeutic strategies.

## 2 EXOSOMAL ncRNAs: BIOGENESIS AND BIOLOGICAL FEATURES

### 2.1 Biogenesis of exosomes

**FIGURE 1:**
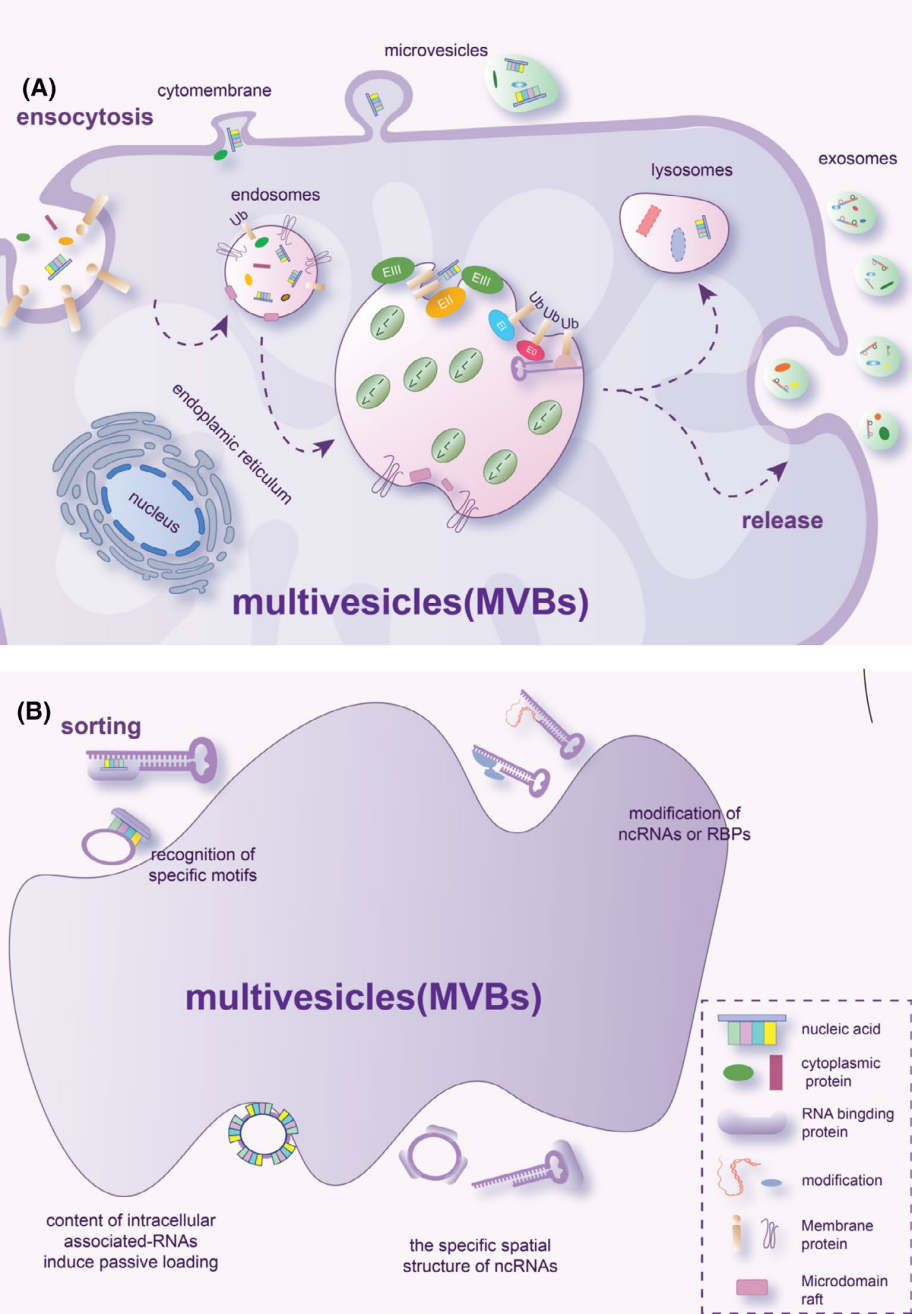
Schematic representation of exosome biogenesis and potential mechanisms for ncRNA sorting. (A) Proteins and nucleic acids are secreted into extracellular vesicles through the budding and fission of the cell membrane, while others enter the cell via the endocytosis pathway, forming endosomes that mature into multivesicular bodies (MVBs). Within MVBs, membrane invagination, budding, and fission lead to the formation of intraluminal vesicles (ILVs) through ESCRT‐dependent and ESCRT‐independent mechanisms. ILVs can either fuse with lysosomes for cargo degradation or dock with the cell membrane to be released as biological exosomes. (B) During ILV formation, specific non‐coding RNAs are selectively sorted into ILVs through four possible mechanisms: (1) recognition of specific motifs on non‐coding RNAs by RBPs; (2) modification of ncRNAs or RBPs, facilitating their incorporation into ILVs; (3) intracellular associated‐RNA content influencing the sorting of ncRNAs into ILVs; and (4) the unique spatial structure of ncRNAs affecting their ability to enter ILVs.

Exosomes were first reported by Johnstone and Stahl in 1983.^24^ The biogenesis of exosomes is a complex, as it involves the formation of bilayer lipid‐bound vesicles (30–150 nm) through the inward budding of endosomes, ultimately leading to the creation of multivesicular bodies (MVBs) as shown in Figure 1A.^25^ The formation of MVBs signifies the transition of early endosomes into late endosomes. This transformation is accompanied by a cargo sorting process that selectively recruits molecular contents, such as proteins, nucleic acids, and metabolites, into intraluminal vesicles (ILVs).^26^ These molecular contents are released into the extracellular space and body fluids by almost every cell, with their expression varying based on the metabolic state of the releasing cells.

The biogenesis of exosomes involves a series of sequential molecular processes, with the endosomal sorting complex required for transport (ESCRT) machinery, including ESCRT‐0, ESCRT‐I, ESCRT‐II, ESCRT‐III, and associated proteins such as vacuolar protein sorting‐associated protein 4A (VPS4A), tumor susceptibility gene 101 protein (TSG101), and ALG‐2‐interacting protein X (ALIX). This system is crucial for the formation of intraluminal vesicles (ILVs).^27^ The ESCRT‐0 ubiquitin‐binding subunits facilitate the sequestration and recognition of ubiquitinated cargo proteins within endosomal membrane domains.^28^ ESCRT‐I and ESCRT‐II can induce ILV budding, while ALIX recruits ESCRT‐III to accelerate membrane pulling, spiral formation, and complete budding.^29^ Moreover, VPS4A and TSG101 play crucial roles in exosome biogenesis through the ESCRT‐dependent pathway.^30^ The ESCRT‐III complex dissociates from the MVB membrane after ILV formation, with sorting protein VPS4A providing the necessary energy for this process.^28^ However, the depletion of ESCRT complexes does not fully inhibit exosome generation, suggesting the presence of an ESCRT‐independent pathway.^31^ Tetraspanins, particularly CD9, CD63, and CD81, are primarily involved in ESCRT‐independent cargo sorting.^32^ Additionally, ceramide is abundant in exosomes, and the activity of sphingomyelinase influences ILV formation.^9^ Therefore, both ESCRT‐dependent and ESCRT‐independent pathways are crucial for exosome production and cargo selection. The synthesized MVBs fuse with the cell membrane to secrete extracellular vesicles into the extracellular space. The trafficking and fusion of MVBs toward the plasma membrane are facilitated by two protein families: the Ras‐associated binding (RAB) protein family and the soluble NSF attachment protein receptor (SNARE) family.^33^ RAB, a GT3 protein, interacts with the cytoskeleton and facilitates intracellular vesicle translocation and extracellular vesicle secretion by placing MVB on the cell membrane, while the SNARE complex facilitates the fusion of the cell membrane and MVB.^34^

### 2.2 Sorting mechanisms of ncRNAs

The transcriptomes of various cell types are partially reflected in the RNA cargo of their extracellular vesicles. However, the RNA profiles of these vesicles differ markedly from those of their cells of origin, indicating that specific RNA species are selectively incorporated into extracellular vesicles.^35^, ^36^ The exact mechanism by which ncRNA selectively enters extracellular vesicles is unknown in eukaryotic cells.^37^ In the following sections, we will focus on recent research into the sorting mechanisms of ncRNAs (Figure 1B).

#### 2.2.1 RNA motif‐dependent RNA‐protein mediated ncRNA sorting

The isolation and regulation of ncRNAs are influenced by characteristic motifs, which are specific sequence or structural features that allow RNA‐binding proteins (RBPs) to recognize and bind to these ncRNAs. These motifs often include particular sequences or secondary structures in RNA molecules, such as stem‐loop or pseudoknot structures. To identify novel RNA‐protein interactions, including RNA‐binding motifs recognized by RBPs and their ncRNA targets, various high‐throughput methods have been developed, particularly in the field of RNA‐binding proteomics.^38^ For example, proteomic analysis reveals a specific binding between heterogeneous nuclear ribonucleoprotein A2B1 (hnRNPA2B1) and extracellular vesicle miR‐198, regulating miRNA loading into microvesicles within exosomes.^39^ hnRNPA2B1, a ubiquitously expressed RBP, can specifically bind miRNAs through the recognition of a short nucleotide sequence, GGAG (named EXO motifs GGAG), and control their loading into exosomes.^39^, ^40^ Unexpectedly, hnRNPA2B1 can negatively regulate the exosomal sorting of ncRNAs, preventing the exosomal export of miR‐503 in endothelial cells due to its high affinity for miR‐503.^41^ RBPs primarily regulate the sorting of exo‐ncRNAs by binding to specific motifs on ncRNAs. Additionally, hnRNPA2B1 has been found to regulate lncRNAs by interacting with specific motifs (GGAG/CCCU) within the 1930–1960‐nt region and stem‐loop structures.^42^ In addition, miR‐133 was selectively sorted into H/R‐induced EPC‐derived exosomes via YBX‐1, enhancing fibroblast angiogenesis and promoting endothelial‐to‐mesenchymal transition (EndoMT).^43^ Recent discoveries have shown that circulating Ago2 complexes stabilize plasma miRNAs via the KRAS‐MEK‐ERK signaling pathway, safeguarding miRNAs within exosomes from RNase degradation.^44^ Considering that the RNA‐binding ubiquitin E3 ligase MEX3C associates with Ago2 and the adaptor‐related protein complex 2 (AP‐2), both involved in miRNA sorting, miR‐451a is specifically sorted into exosomes via a ceramide‐dependent pathway, facilitated by MEX3C's C‐terminal RING finger domain and hnRNP K homology domain.^45^, ^46^ In general, the involvement of specific ncRNA motifs binding to RBPs in exosomal ncRNA sorting has been confirmed across various cell types.

#### 2.2.2 Relationship between RNA posttranscriptional modification and RNA sorting

An important mechanism involves posttranscriptional modifications of ncRNA, such as phosphorylation, methylation, uridylation, or 3′‐terminal adenylation, alternative splicing, or terminal nucleotide deletion that guide their sorting into exosomes.^47^ For instance, Koppers et al. reported that 3’‐end adenylation of specific miRNA species is associated with their relative enrichment in cells compared to exosomes, while 3’‐end uridylated isoforms are overrepresented in exosomes, as demonstrated in naturally occurring exosomes isolated from human urine samples. The results suggest that posttranscriptional modifications, notably 3’ end adenylation and uridylation, have opposing effects that may contribute to the selective sorting of ncRNAs into exosomes. One of the key RNA methylation types, m5C, is regulated by NSun2, which methylates cytosine‐5 in most tRNAs, as well as in other ncRNAs and mRNAs. The molecular role of NSun2 varies depending on the class of RNA it modifies. Research suggests that m5C is involved in RNA sorting into exosomes, a process where cells secrete specific RNA molecules for specific biological functions. Islayem et al. confirmed that RNA posttranscriptional modifications like m5C and m6A are recognized by exosomes to be sorted inside them, are strengthened, and considered valid.^48^

#### 2.2.3 Regulating the content of intracellular RNA and sorting ncRNA into exosomes

Exosomes facilitate cell communication, playing a critical role in tumorigenesis by enabling interactions between tumor cells and non‐tumor cells, which promotes tumor growth, survival, progression, angiogenesis, and metastasis.^49^ Numerous studies have demonstrated that exosomal contents serve as cancer biomarkers, aiding in early diagnosis and assessing cancer progression.^49^ Downregulation or upregulation of miRNAs, which are crucial members of the short ncRNA family, play a significant role in cancer progression by either inhibiting or promoting tumor growth through the modulation of tumor suppressor genes or oncogenes.^49^ It has been demonstrated that exosomal miRNA‐9 promotes tumor angiogenesis by activating the activators of transcription pathway activating and the Janus kinase/signal transducers through the downregulation of cytokine signaling 5.^50^ Another study demonstrated that miRNA‐105 promotes metastasis and induces vascular leakiness, while its inhibition in highly metastatic tumors alleviates these effects.^51^ In addition, it has been shown that ncRNAs can interact with their target RNAs to regulate the cellular levels of either themselves or their targets, before being sorted into exosomes either passively or actively. Squadrito et al. suggested that the physiological activation or artificial overexpression of miRNA target sequences (mRNA) in macrophages contributes to the enrichment of corresponding miRNAs in exosomes and P‐bodies. This mechanism may reduce miRNA loading in producer cells, potentially altering miRNA activity and helping to maintain cellular homeostasis.^52^

#### 2.2.4 Secondary/tertiary structure of ncRNAs regulates their sorting

The secondary and tertiary structures of RNA, particularly in ncRNAs due to their greater protein‐binding capacity, can influence their sorting into exosomes.^44^ Shurtleff et al. demonstrated that YBX1 interacts with miR‐223 based on its structure rather than a specific motif sequence, recognizing it through its internal cold shock domain, which forms a hairpin‐loop secondary structure, thereby guiding its localization into exosomes.^53^ Furthermore, other studies have shown that circRNAs can be sorted into exosomes due to their distinct circular tertiary structures.^54^

## 3 ROLES OF EXOSOMAL ncRNAs IN THE BC MICROENVIRONMENT

### 3.1 Promote inflammatory microenvironment

**FIGURE 2:**
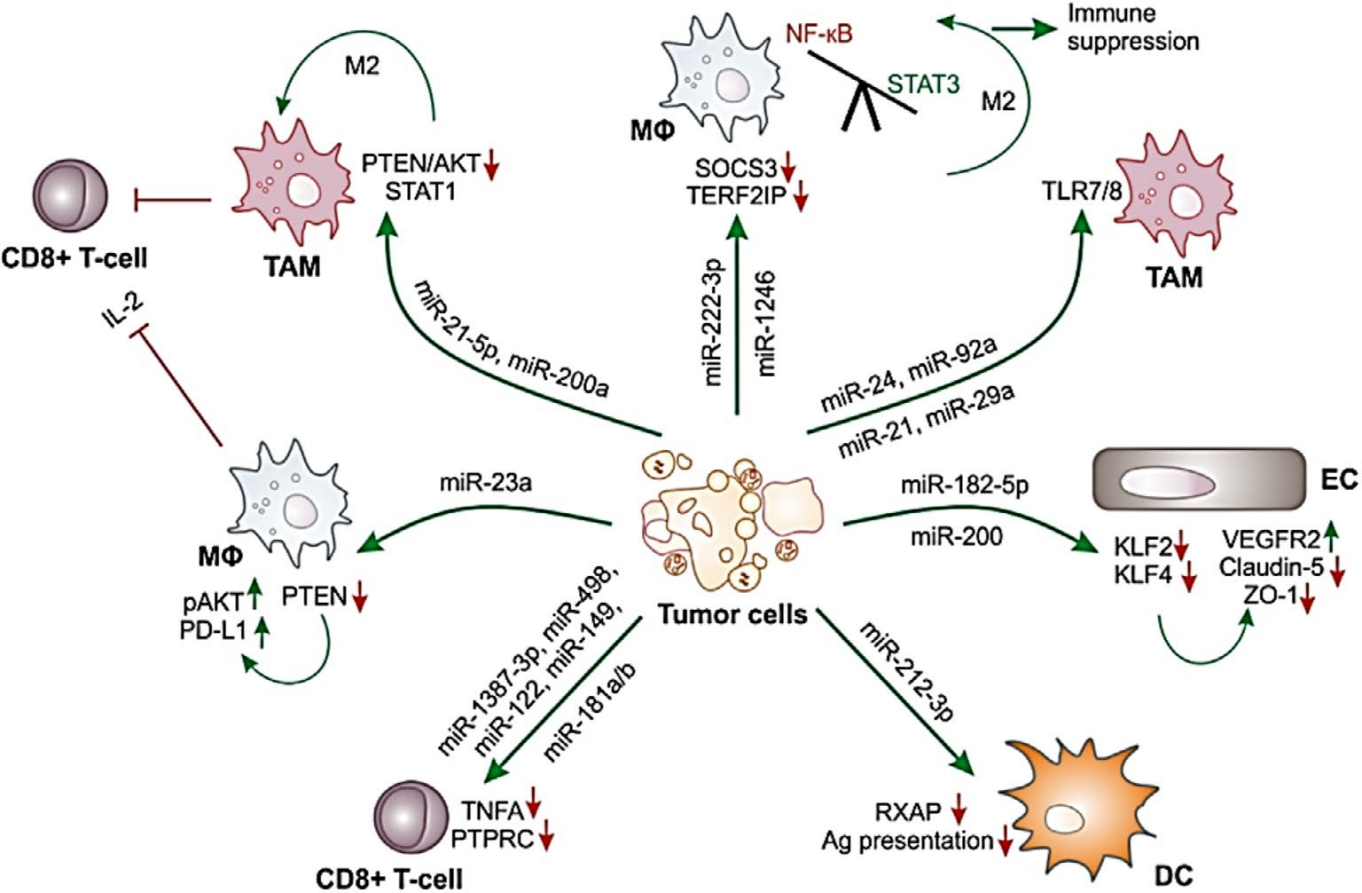
Exosomal miRs in the tumor microenvironment. Reproduced with permission.^60^ Copyright 2022, Licensee MDPI, Basel, Switzerland.

The tumor immune microenvironment (TIME) plays a pivotal role in determining tumor progression. Initially, the immune system may act to eliminate tumors through anti‐tumor immunity, but in later stages, cancers can manipulate the TIME into an immunosuppressive state, helping them evade immune surveillance and promote tumor growth.^55^, ^56^ Persistent low‐grade inflammation in the TIME supports tumor growth and migration by creating conditions that are favorable for cancer progression. Tumor‐derived factors contribute to this altered microenvironment, ensuring that inflammation persists.^57^ For example, upregulation of nuclear factor κB (NF‐κB) and STAT1induces the M1 phenotype, whereas the activation of STAT6 and STAT3 stimulates M2 polarization.^58^ Epigallocatechin gallate enhances exosomal miR‐16 secretion in 4T1 BC cells. This miR‐16 suppresses CSF‐1 and CCL‐2 expression, reprogramming tumor‐associated macrophages (TAMs) via reduced TGF‐β and IL‐6 levels and elevated TNF‐α through the NF‐κB pathway. Consequently, macrophage infiltration and M2 polarization are reduced.^59^ Casadei et al. reported that reported that exosomes derived from liposarcoma cells are enriched with miR‐24 and miR‐92a. These miRNAs promote the production and release of pro‐inflammatory IL‐6 from tumor‐associated macrophages (TAMs) through the activation of toll‐like receptor (TLR)7/8 and NF‐κB signaling pathways (Figure 2).^60^ The released IL‐6 subsequently enhances the proliferation, migration, and invasion capabilities of liposarcoma cells. Moreover, ncRNAs derived from cancer cells can interact with specific biomolecules, modulating cytokine and chemokine release to exert regulatory effects on macrophages.^61^ In one study, exosomal miR‐33 and miR‐130 secreted by BC cells were delivered to IL‐4‐induced TAMs or macrophages, leading to the elevation of M1 markers (CD80, TRF5, and MCP1) and cytokines (TNF‐α and IL‐1β). This suggests that these miRNAs induced the repolarization of macrophages from the M2 phenotype to the M1 phenotype.^62^

### 3.2 Promote the formation of a hypoxic microenvironment

Hypoxia, a key characteristic of solid tumors, promotes tumor progression, metastasis, and angiogenesis, potentially through exosome‐mediated signaling pathways.^63^ For example, King et al. demonstrated that hypoxia enhances the release of exosomes by breast cancer cells, promoting their survival and invasion, with this hypoxic response potentially being mediated by hypoxia‐inducible factor‐1α.^64^ The hypoxically regulated miR‐210 was identified to be present at elevated levels in hypoxic exosome fractions. The study of Wang et al. suggested that hypoxia upregulates HIF‐dependent RAB22A gene expression in advanced breast cancer, thereby enhancing cancer cell invasion and promoting micro‐vesicle generation.^65^ Feng et al. demonstrated that breast cancer cells secrete exosomal miR‐22‐3p, which mediates tumor vessel abnormalization by suppressing transgelin, thereby promoting tumor budding and breast cancer progression in vivo.^66^ Pan et al. found exosomal miR‐145 in MDA‐MB‐231 cells targeted IRS1 and inhibited the angiogenesis of HUVECs by regulating IRS1/PI3K/Akt/mTOR and IRS1/Raf/ERK pathways.^67^ Notably, stromal interaction molecule 1 (STIM1), a transmembrane protein located in the endoplasmic reticulum, was shown to reduce BC exosomal miR‐145, thereby promoting angiogenesis and migration. Given the emerging role of tumor cell‐derived exosomes in tumor progression, understanding the link between hypoxia and exosome biogenesis is of significant importance.^26^

### 3.3 Regulating the immunosuppressive microenvironment

Several studies have reported the impact of breast cancer cell‐derived exosomes on the immune system, particularly their interactions with T cells, dendritic cells (DCs), macrophages, and regulatory T cells.^25^ They can function as paracrine messengers within the immune system, transporting either immunosuppressive agents or pro‐inflammatory signals.^68^, ^69^ The inflammatory environment surrounding BC promotes its expansion by evading the immune system, leading to distant metastasis, a major cause of BC‐related mortality.^70^, ^71^ Exosome‐mediated immune suppression was demonstrated in a study where exosomes from TS/A murine mammary tumors partially induced IL‐6 mRNA, inhibiting the differentiation of myeloid precursors into DCs.^25^ Tumor‐derived exosomes can alter the function of DCs within tumors, lymph nodes, and metastatic sites. These exosomes suppress effector T‐cell responses by promoting the differentiation of regulatory T ells, which contributes to cancer metastasis and progression.^72^ Another study found that injecting TS/A cell‐derived exosomes into a mouse model led to the accumulation of myeloid‐derived suppressor cells within the tumor, which in turn suppressed T‐cell responses.^73^ BC exosomes modulated Let‐7i, miR‐142, and miR‐155, synergistically inducing DC maturation and facilitating tumor immune evasion.^74^ Meanwhile, BC exosomes carrying programmed death‐ligand 1 (PD‐L1) exhibited strong immunosuppressive effects within the BC‐TME. TGF‐β was found to promote exosomal PD‐L1, leading to CD8 T‐cell dysfunction by attenuating the phosphorylation of Src family proteins in activated CD8 T cells. This finding highlights the role of TGF‐β in enhancing the immunosuppressive activity of tumor‐derived exosomal PD‐L1, thereby contributing to immune escape.^75^ These results all confirmed the significance of the roles of tumor‐derived exosomes in communicating with immune cells, involved in DC differentiation and maturation, CD8 T‐cell dysfunction, and regulatory immune cells like MDSCs and Tregs. More comprehensive proteomic and RNA profiling of BC‐derived exosomes could further elucidate key exosomal components that drive immune regulation.

### 3.4 Reshaping the fibrotic microenvironment

**FIGURE 3:**
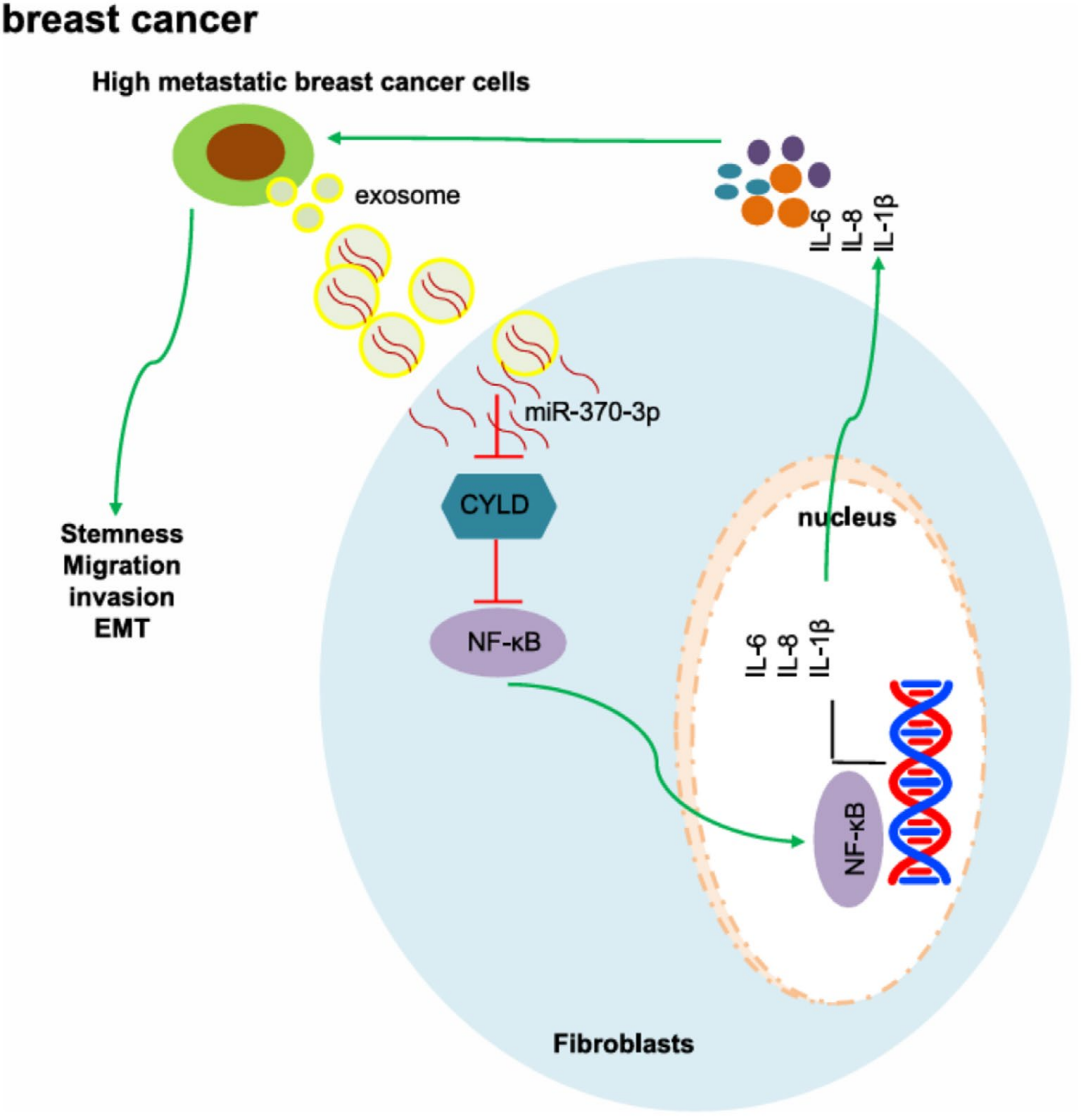
Schematic diagram of the mechanism of miR‐370‐3p‐containing EVs released from breast cancer cells in breast cancer. Highly metastatic breast cancer cell‐derived EVs transfer miR‐370‐3p from breast cancer cells to fibroblasts. The transferred miR‐370‐ 3p targets CYLD and activates the NF‐ κB signaling pathway, thus stimulating the expression of IL‐6, IL‐8, and IL‐1β, promoting the stemness, migration, invasion, and EMT of breast cancer cells. Reproduced with permission.^81^ Copyright 2021, Federation of American Societies for Experimental Biology.

Cancer‐associated fibroblasts (CAFs) are crucial in the tumor microenvironment and significantly contribute to the occurrence and development of tumors.^76^ CAFs do not function in isolation around tumors but engage in dynamic interactions that promote tumor growth and survival. Studies suggest that exosomal ncRNAs play a critical role in this crosstalk, reshaping the fibrotic microenvironment by mediating communication between BC cells and CAFs. For example, exosomes transport miRNAs to normal fibroblasts (NFs), transforming them into CAFs. In 2016, Baroni et al. documented that exosomal miR‐9 from BC cells induced CAF‐like properties in human breast fibroblasts.^77^ In 2019, In 2019, Vu et al. demonstrated that miR‐125b from breast cancer cells facilitated the conversion of NFs into CAFs.^78^ Meanwhile, Chatterjee et al. reported a significant upregulation of miR‐222 in CAFs compared to NFs, with miR‐222 overexpression being sufficient to induce CAF‐like characteristics in NFs.^79^ In the 2020 study by Yang et al., exosomal miR‐146a from BC cells accelerated the transformation of NFs to CAFs by targeting thioredoxin interacting protein.^80^ In 2021, Ren et al. proved that exosomal miR‐370‐3p activated fibroblasts, enhancing their stemness, migration, and invasion of cancer cells, as shown in Figure 3.^81^

### 3.5 Impact of ncRNA‐exosome on multidrug resistance in tumors

The resistance of BC cells to therapeutic agents continues to be a significant challenge in the treatment of BC patients.^4^ Some factors such as exosomes, soluble receptors, and cytokines have been identified as key contributors to the development of drug resistance. However, the exact mechanisms underlying the drug resistance are still unknown.^82^, ^83^ There is growing evidence highlighting the emerging roles of exosomes, particularly exosomal ncRNAs, in tumor initiation, metastasis, drug resistance, migration, invasion, angiogenesis, and proliferation.^20^ For example, Wei et al. demonstrated that exosomal miR‐221/222 enhanced tamoxifen resistance in recipient ER‐positive BC cells, while anti‐miR‐221/222 effectively blocked the spread of this resistance.^84^ Similarly, exosomes from MCF‐7/Adr and MCF‐7/Doc cells were found to mediate resistance through the intercellular transfer of specific miRNA cargoes, particularly miR‐222.^85^ These results provided a new perspective on enhancing tamoxifen efficacy in BC patients. Pan et al. further reported that exosomal miR‐221‐3p from BC cells could promote resistance to ADR by modulating the PIK3R1‐dependent PI3K/AKT signaling pathway, both in vitro and in vivo.^86^ Trastuzumab has significantly improved the clinical outcomes for HER2‐positive BC patients; however, many patients eventually develop resistance to the therapy, leading to disease progression. The dysregulation of lncRNAs is believed to play a critical role in the development of trastuzumab resistance. In one study, exosomal lncRNA‐SNHG14 was found to be upregulated in the serum of patients resistant to trastuzumab.^87^ This lncRNA promoted trastuzumab resistance by targeting the apoptosis regulator Bcl‐2/Bax. The exosomal lncRNA‐SNHG14 promoted the effect of trastuzumab via targeting the apoptosis regulator Bcl‐2/Bax. In the in vitro assay, Chen et al. suggested that exosomal lncRNA AGAP2‐AS1 could reduce trastuzumab‐induced cell death in sensitive cells.^88^ In addition, it is well‐established that circRNAs play a crucial role in regulating key signaling pathways in breast cancer, particularly in chemoresistant cell lines and clinical samples. For instance, Yang et al. reported that circ‐ABCB10 contributes to paclitaxel resistance in BC cells by upregulating DUSP7 through capturing let‐7a‐5p.^89^ circBMPR2 may act as a sponge for miR‐553, alleviating its inhibition of ubiquitin‐specific protease 4 (USP4), thereby suppressing BC progression and resistance to tamoxifen.^90^

## 4 ROLE OF EXOSOMAL ncRNA IN DISTANT METASTASIS

### 4.1 Role of exosomal ncRNA in epithelial‐mesenchymal transition

EMT is a widely accepted mechanism underlying distant metastasis in epithelial cancers, including BC.^91^ EMT is characterized by the disruption of intracellular tight junctions and the loss of cell–cell adhesion, leading to a transition from epithelial traits to a mesenchymal phenotype. This process results in enhanced cell self‐renewal capabilities and the emergence of heterogeneous subpopulations.^91^ These features improve cell motility, allowing cells to release from the parental epithelial tissue site and reconstitute metastatic colonies at distant sites.^92^, ^93^ In recent years, exosomal ncRNAs have been reported in the regulation of EMT in BC.^94^ For example, Wang et al. found exosomal microRNA‐181d‐5p promotes EMT in breast cancer by regulating the CDX2/HOXA5 axis, highlighting the crucial role of microRNAs in tumor invasion and metastasis (M.^95^). miR‐218, a tumor‐suppressor miRNA, is downregulated in association with EMT and angiogenesis. Shojaei S et al. suggest that ADMSC‐derived exosomes can restore miR‐218 levels in breast cancer cells and that miR‐218 can inhibit breast cancer progression by simultaneously targeting both angiogenesis and EMT. In an in vitro study, treating MDA‐MB‐231 cells with ADMSC‐exosomes/miR‐218 increased the expression of the epithelial marker CDH1 and decreased the expression of the mesenchymal marker CDH2, while also reducing the viability, motility, invasion, and angiogenic capacity of BC cells.^96^

### 4.2 Role of exosomal ncRNA in pre‐metastatic niche

**FIGURE 4:**
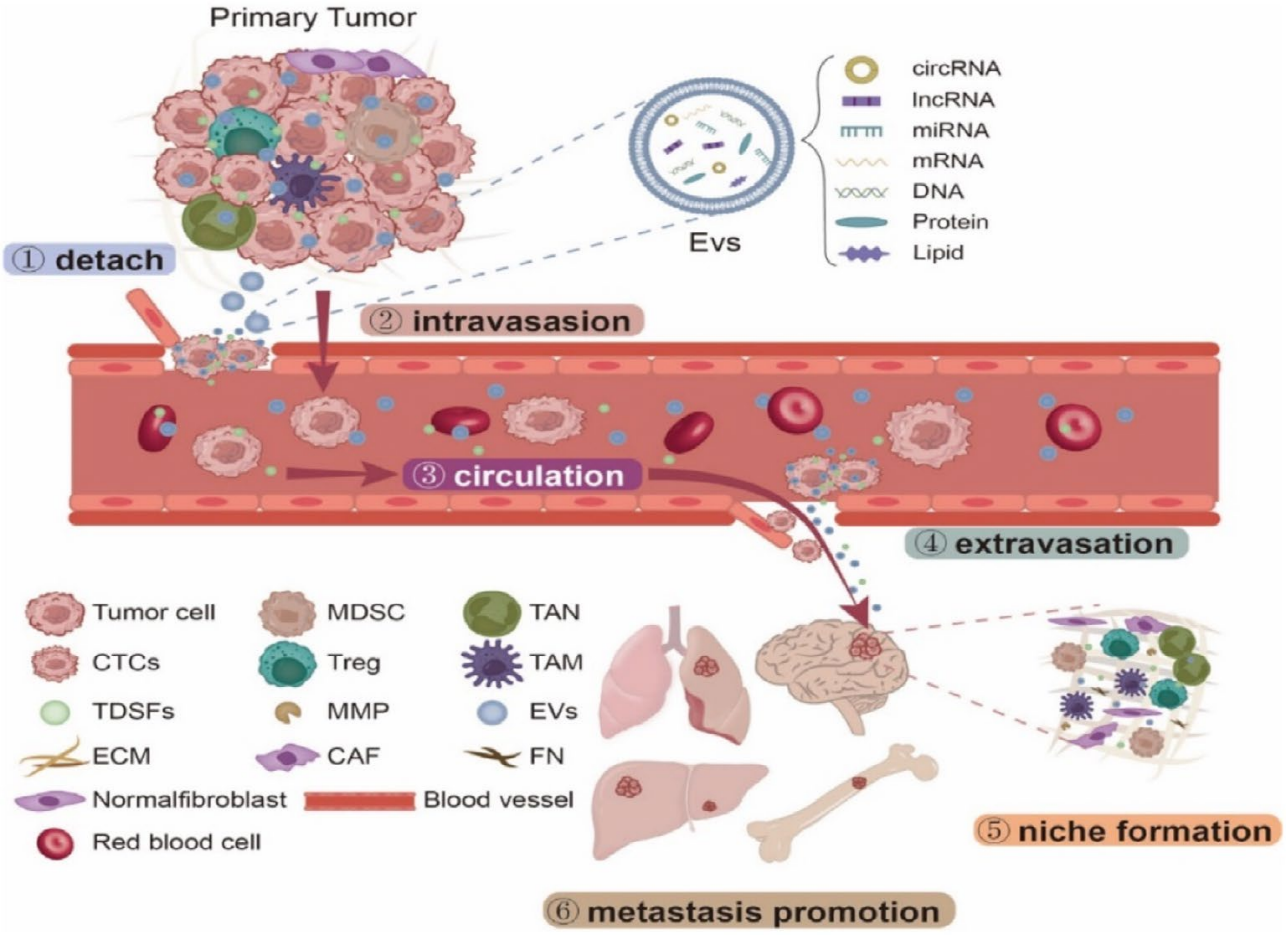
Exosomal ncRNAs are involved in multiple stages of tumor‐distant metastasis. These stages include the detachment of tumor cells from the primary site, penetration through the basement membrane, entry into circulation, and induction of PMN formation, ultimately facilitating the establishment of metastases in target organs. Reproduced with permission.^97^ Copyright 2022, Licensee MDPI, Basel, Switzerland.

Metastasis is a pivotal stage in tumor progression, presenting a significant challenge for clinical therapy and a leading cause of patient mortality. Numerous studies have confirmed that distant tumor metastasis relies on the formation of a pre‐metastatic niche (PMN).^97^ Recent studies have highlighted the critical role of exosomes in the formation of the PMN, as shown in Figure 4. Exosomal ncRNAs contribute to PMN formation and distant tumor metastasis by suppressing anti‐tumor immune responses, fostering an inflammatory environment, inducing vascular permeability and angiogenesis, and reprogramming the microenvironment.^97^ Alveolar epithelial type II cells internalized exosomal miR‐200b‐3p, leading to the upregulation of chemokine ligand 2, S100A8/9, matrix metalloproteinase 9, and colony‐stimulating factor 1. This upregulation facilitated the recruitment of myeloid‐derived suppressor cells and promoted the formation of an inflammatory PMN.^98^ This study demonstrated how exosomal ncRNAs contribute to the upregulation of pro‐inflammatory cytokines, fostering the formation of an inflammatory microenvironment at distant tumor sites and playing a critical role in initiating PMN. Lin28B reduced let‐7 levels in exosomes, modulating the expression of CXCL, IL‐10, and IL‐6 in neutrophils, leading to an immunosuppressive PMN and facilitating lung metastasis of BC.^99^ This result suggested that ncRNAs carried by exosomes can inhibit immune system functions in various ways, facilitating tumor immune escape and playing a crucial role in PMN formation.^97^ In addition, BC‐derived exosomes carry miR‐105, which downregulates ZO‐1, causing endothelial cell monolayer barrier function disruption, increasing vascular permeability, and promoting tumor metastasis within the pre‐metastatic niche.^51^

## 5 ROLE OF EXOCRINE ncRNAs IN THE DIAGNOSIS AND PROGNOSIS OF BC

### 5.1 Role of exosomal ncRNA in the diagnosis

Despite significant advances in early detection, diagnosis, and therapy, BC remains one of the leading causes of cancer‐related deaths in women, primarily due to poor prognosis resulting from tumor metastasis and recurrence.^100^ Over the past decades, numerous studies have attempted to elucidate the mechanisms driving BC proliferation, oncogenesis, and metastasis.^101^ However, these mechanisms remain incompletely understood. Identifying key molecules and pathways is crucial for developing predictive and diagnostic biomarkers, as well as innovative treatments to combat BC.^20^ Exosomes can be isolated from patient samples such as ascites fluid, urine, pleural effusions, and serum. Assessing these exosomes offers a non‐invasive approach to developing diagnostic methods, enabling the monitoring of disease progression, therapeutic efficacy, and resistance mechanisms, as their cargo reflects the tumor's status, including stage and subtype.^25^ Exosomes from breast milk and salivary glands have shown promise as potential biomarkers for BC detection. Notably, breast milk exosomes with elevated levels of TGF‐β2 have been positively correlated with an increased risk of BC.^102^, ^103^ Numerous studies have demonstrated differential expression of exosomal miRNAs between BC patients and healthy individuals (Table 1). Specifically, miR‐155 is upregulated in the tumor cells of breast cancer patients, particularly those with TNBC and early‐stage disease. As a result, plasma miR‐155 could serve as a non‐invasive biomarker for the early detection of BC.^104^ A study found that elevated levels of exosomal miRNA‐21, miRNA‐222, and miRNA‐155 were significantly associated with the detection of circulating tumor cells, suggesting that combining exosomal miRNA analysis with CTC detection could enhance the diagnosis and prognosis for patients undergoing neoadjuvant therapy.^105^ In a review of Sun et al., exosomal lncRNA in the serum of colorectal cancer (CRC) patients, highlighting its potential as both a diagnostic and therapeutic tool for CRC.^106^ Du et al. analyzed the expression and function of lncRNA PCAT‐14 in hepatocellular carcinoma (HCC), finding it to be overexpressed in HCC patients.^109^ In addition, circRNAs have emerged as specific biomarkers for certain cancers, with research primarily focusing on their role in diagnosing lung cancer and BC.^110^, ^111^

**TABLE 1:** Various exosomal ncRNAs and their roles in the diagnosis and prognosis of BC.

Type of ncRNAs	Expression	Function/role	Ref.
miRNA‐155	Upregulation	As a non‐invasive biomarker	Gao et al. [104]
miRNA‐21	Upregulation	As both a diagnostic and therapeutic tool	Rodriguez‐Martinez et al. [105]
miRNA‐222	Upregulation		
ncRNA PCAT‐14	Upregulation	As specific biomarkers for BC	Sun et al. [106]
miR‐148a	Deregulation	As a promising diagnostic and prognostic biomarker	Li et al. [107]
lncRNA SUMO1P3	Upregulation	As a promising prognostic biomarker	Zhan et al. [108]

### 5.2 Role of exosomal ncRNA in the prognosis of BC

Early detection, therapy, and metastasis monitoring are crucial for a favorable prognosis, while conventional diagnostic programs like breast X‐rays and ultrasounds often cause radioactive or invasive damage to patients with limited accuracy.^112^, ^113^ Liquid biopsy, as a non‐invasive method, offers the convenience of repeated sampling for recurrence monitoring, metastatic assessment, and clinical cancer prognosis. Aberrant profiles of ncRNAs in exosomes released into circulation, particularly in plasma and serum, present promising candidate biomarkers for BC detection through liquid biopsy (Table 1).^20^ For example, serum exosomal miR‐148a has emerged as a promising biomarker for BC diagnosis and prognosis prediction. Li et al. reported that serum exosomal miR‐148a levels were significantly reduced in BC patients, with lower levels correlating with an unfavorable prognosis.^107^ Moreover, exosomal lncRNAs have been identified as crucial players in BC tumorigenesis, with their abnormal expression in serum potentially serving as prognostic biomarkers for BC.^114^, ^115^ Small ubiquitin‐like modifier 1 pseudogene 3 (SUMO1P3) is a newly identified lncRNA that is aberrantly expressed in various cancers, including BC.^108^, ^116^, ^117^ Na‐Er et al. demonstrated that serum exosomal lncRNA SUMO1P3 is significantly upregulated in TNBC, suggesting it as a promising prognostic biomarker for improving individual patient outcomes.

## 6 ROLE OF EXOSOMAL ncRNA IN THE TREATMENT OF BC

### 6.1 Exosomal ncRNAs as inhibitors for BC

ncRNAs can function as both oncogenes and tumor suppressors, leading to the abnormal inhibition or degradation of target mRNAs. As a result, they present themselves as both direct therapeutic targets and promising candidates for cancer treatment.^118^ Utilizing miRNA‐based therapeutics provides dual benefits. First, as naturally occurring molecules in human cells, miRNAs have intrinsic mechanisms for processing and targeting, unlike synthetic chemotherapy agents or antisense oligonucleotides (ASOs). Second, miRNAs can regulate multiple genes within a single pathway, enabling a more comprehensive and precise therapeutic response.^118^ For instance, exosomal miR‐23b derived from bone marrow MSCs has been found to suppress the MARCKS gene, which encodes a protein that enhances cell cycling and motility. This suppression induces quiescence in BC cells and reduces CD44 expression in BCSCs, thereby promoting dormancy.^119^ Li et al. determined the role of miR‐770 in the regulation of chemo‐resistance and metastasis of TNBC, which was mediated by exosomes.^120^ The results demonstrated that miR‐770 can suppress doxorubicin resistance and metastasis in TNBC cells, offering new insights into the mechanisms underlying chemo‐resistance and metastasis, and providing a potential prognostic marker for TNBC. In addition, the overexpression of miR‐451 directly targets YWHAZ to inhibit β‐catenin expression, thereby increasing BC cell sensitivity to paclitaxel.^121^

### 6.2 Exosomal ncRNA as a targeted drug carrier

**FIGURE 5:**
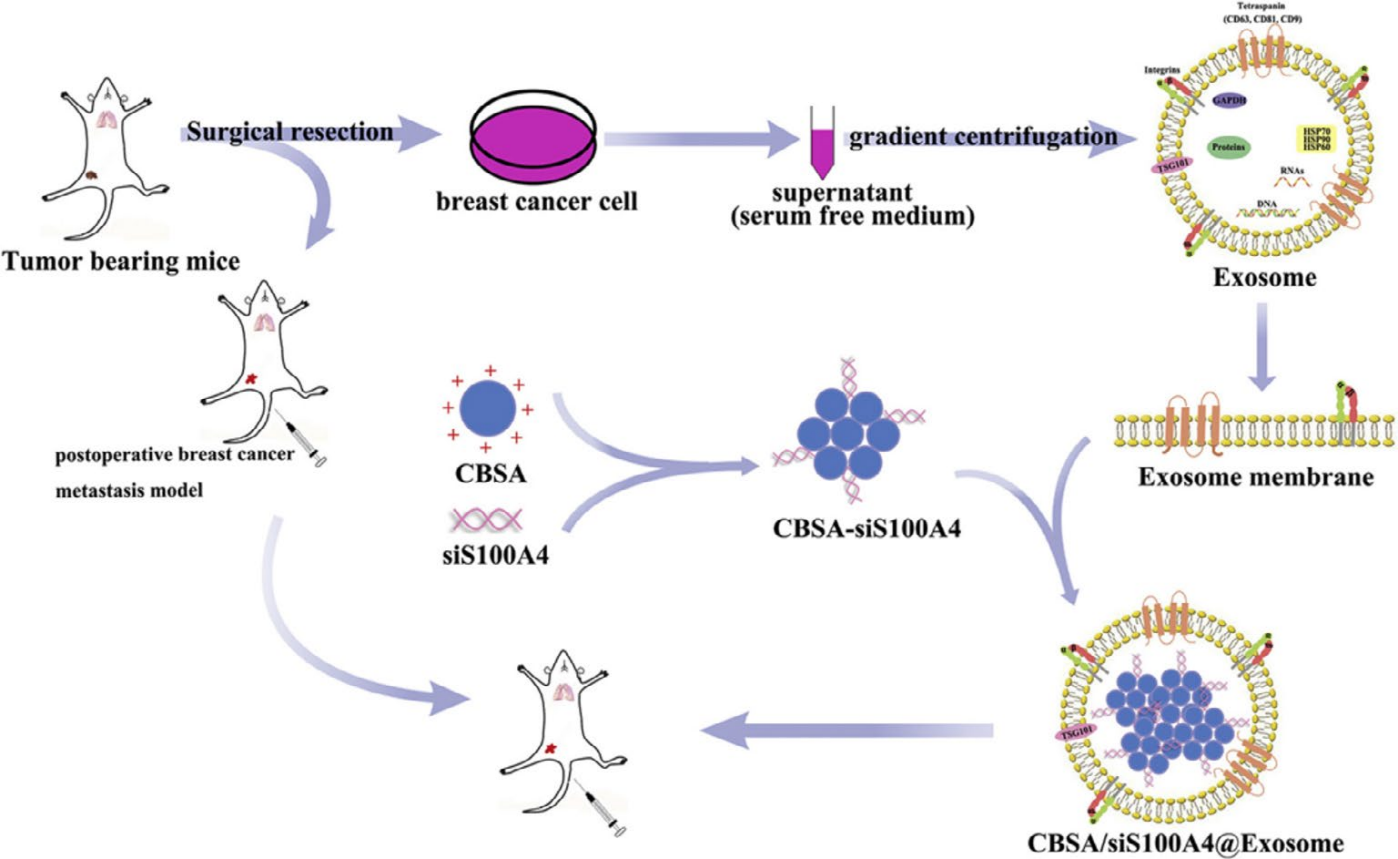
Schematic illustration of exosome‐mediated siRNA delivery for the suppression of postoperative BC metastasis. Reproduced with permission.^124^ Copyright 2020, Elsevier B. V.

Exosomes, natural nanovesicles, have emerged as a novel mode of intercellular communication due to their ability to transmit essential cellular information. They can be engineered for enhanced delivery and targeting capabilities. Their advantages include a sophisticated communication system that enables precise and direct cellular delivery of diverse cargo molecules, including nucleic acids, along with limited toxicity, excellent biocompatibility, and adjustable circulation stability.^122^, ^123^ Zhao et al. developed biomimetic nanoparticles, specifically cationic bovine serum albumin (CBSA) conjugated with siS100A4 and coated with an exosome membrane (CBSA/siS100A4@Exosome), to enhance drug delivery aimed at suppressing postoperative lung metastasis in TNBC, as shown in Figure 5.^124^ The study utilized exosome membrane‐based nanoparticles to deliver therapeutic siS100A4 to the lungs, demonstrating effective cancer prevention and treatment in a mouse model of postoperative lung metastasis. Gong et al. developed a strategy to isolate exosomes with enhanced affinity for integrin αvβ3 by modifying their membranes with a disintegrin and metalloproteinase 15 (A15). These A15‐modified exosomes (A15‐Exo) facilitated the co‐delivery of therapeutic doses of doxorubicin (Dox) and cholesterol‐modified miRNA 159 (Cho‐miR159) to TNBC cells, both in vitro and in vivo.^125^ In vitro, A15‐Exo co‐loaded with Cho‐miR159 and Dox demonstrated synergistic therapeutic effects in MDA‐MB‐231 cells. In vivo, the vesicular delivery of miR159 and Dox effectively silenced the TCF‐7 gene and enhanced anticancer efficacy, all while avoiding adverse effects. Therefore, these results demonstrate the synergistic efficacy of co‐delivering miR159 and Dox by targeted Exo for TNBC therapy. Another study by O'Brien et al. explored the in vivo delivery of the tumor‐suppressor miR‐379 for breast cancer therapy using extracellular vesicles with established tumor‐homing capabilities.^126^ The administration of extracellular vesicle encapsulated miR‐379 significantly reduced tumor formation and growth in T47D BC cells expressing miR‐379. Naseri et al. utilized exosomes derived from bone marrow‐derived mesenchymal stem cells (MSCs) to deliver anti‐miR‐142‐3p. They observed a reduction in miR‐142‐3p and miR‐150 levels and an increase in the transcription of regulatory genes APC and P2X7R in TUBO breast cancer cell lines. The MSC‐derived exosomal system effectively penetrated the tumor site and delivered the inhibitory oligonucleotides, leading to downregulation of the targeted microRNAs.^127^

## 7 CONCLUSIONS AND OUTLOOK

As crucial mediators of cell–cell communication, exosomal ncRNAs serve as versatile carriers with complex roles in breast cancer progression. Once released by parental cells, these ncRNAs are shuttled and transferred to neighboring or distant cells, where they play a role in reprogramming the TME. This review emphasizes that exosomal lncRNAs, miRNAs, and circRNAs are involved in key processes such as proliferation, apoptosis, invasiveness, EMT, and angiogenesis. Through these roles, they contribute to breast cancer proliferation, oncogenesis, drug resistance, and metastasis. Based on specific deregulated expression verified, these exosomal ncRNAs present in blood samples are promising candidates for enhancing early diagnosis, therapeutic monitoring, and personalized prognosis. In addition, exosomal ncRNAs, as exceptional mediators of cell–cell communication, hold promise for targeted delivery in BC treatment. However, despite significant advancements, realizing the full potential of exosomal ncRNAs requires overcoming several challenges. Firstly, the complex role of exosomal ncRNAs in BC progression requires further investigation. As the TME is a complex entity, where various cell types secrete distinct exosomal ncRNAs, it would be valuable to explore which specific factors, secreted by particular cell types, exert the most significant or dominant impact on BC. Given the high genetic heterogeneity of BC tumors, elucidating the pathogenesis of key exosomal ncRNAs is crucial for developing new therapeutic strategies. Secondly, since metastasis is the primary cause of death in BC patients, identifying novel ncRNAs or exosomal ncRNAs to combat metastasis holds significant value. It has been shown that BC‐derived exosomes play a pivotal role in the metastasis and dissemination of primary tumors, influencing everything from oncogenic reprogramming of malignant cells to the formation of the pre‐metastatic niche. These effects are mediated through cell–cell crosstalk, modifying both the local and distant microenvironments in autocrine and paracrine fashions. Identifying key exosomal ncRNAs involved in metastasis could enhance precision medicine strategies for diagnosing and treating BC metastasis.^128^ Thirdly, studies on the asymmetric distribution of ncRNAs between parent cells and their exosomes suggest a selective encapsulation of these regulatory RNAs during exosome biogenesis.^129^ Coincidentally, Ni et al. observed a selective, wave‐like packaging of miR‐16 into exosomes across different BC subtypes, potentially linked to tumor development and progression.^130^ Therefore, while the differentially expressed ncRNAs in exosomes may show varying degrees of difference from their expression levels in parental cells, they still reflect the distinct pathological states of BC. However, the mechanisms underlying the selective packaging of ncRNAs into exosomes require further elucidation. Fourthly, although numerous studies have explored the characteristics of circRNAs in BC, reports on exosomal circRNAs remain limited. Currently, most identified circRNA biomarkers lack the sensitivity and specificity required for clinical application. The biological functions, biogenesis, abundance, and potential sorting mechanisms of exosomal circRNAs, along with their roles in promoting or inhibiting cancer, warrant further exploration. In addition, the lack of an optimal purification technique for isolating exosomes with high purity remains a challenge.^131^ The various isolation and existing separation techniques often yield low quantities of exosomes and are costly for large‐scale production.^132^ To enhance the targetability of exosomes, ligands are chemically conjugated to their surface. The efficacy and safety of these active targeting molecular combinations require thorough examination. In conclusion, we present the latest insights into the roles and mechanisms of exosomal ncRNAs in drug resistance, detection, metastasis, and tumor growth in BC. Further research on exosomal ncRNAs will not only enhance our understanding of their function but also contribute to improved clinical outcomes for individuals with BC.

## AUTHOR CONTRIBUTIONS

Conceptualization, Xiang Li; writing—original draft preparation, Junyi Gong; writing‐review and editing, Yi Zhang; visualization, writing—review and editing, Xiang Ni and Junli Yin; supervision and project administration, Zheng Lv. All authors have read and agreed to the published version of the manuscript.

## FUNDING INFORMATION

The authors received no financial support for the research, authorship, and publication of this article.

## DISCLOSURES

The authors declare that they have no known competing financial interests or personal relationships that could have appeared to influence the work reported in this paper.

### DATA AVAILABILITY STATEMENT

Data sharing is not applicable to this article as no new dates were created nor analyzed in this study.
